# Is there a correlation between -174(G/C) polymorphism of IL-6 gene and the incidence of acute myocardial infarction?

**DOI:** 10.1186/s43141-021-00243-x

**Published:** 2021-09-20

**Authors:** Ingy M. Hashad, Habiba Nosseir, Gamal M. Shaban, Mohamed F. Abdel Rahman, Mohamed Z. Gad

**Affiliations:** 1 Clinical Biochemistry Unit, Faculty of Pharmacy and Biotechnology, the German University in Cairo, Cairo, Egypt; 2 National Heart Institute, Cairo, Egypt; 3 Department of Biology and Biochemistry, School of Life and Medical Sciences, University of Hertfordshire Hosted by Global Academic Foundation, Cairo, Egypt

**Keywords:** Inflammation, SNP, Cardiovascular disease, ELISA

## Abstract

**Background:**

Cardiovascular disease (CVD) remains the major cause of death worldwide. Most CVD can be prevented by addressing risk factors. Acute myocardial infarction (AMI) is an inflammatory disorder characterized by changes in several cytokines including the interleukins (ILs). Studies are running to evaluate the genetic variation in the inflammatory system and their influence on the risk factors for CVD aiming for future prevention of this global disease. The aim of the current study was too investigate the association of -174 (G/C) IL-6 polymorphism with the incidence of AMI in a representative sector of the Egyptian population and to examine the contribution of IL-6, as a biomarker, in the pathogenesis of AMI. Genotyping of -174 (G/C) IL-6 polymorphism was done by polymerase chain reaction-restriction fragment length polymorphism (PCR-RFLP) while IL-6 levels were assayed by ELISA.

**Results:**

The genotype distribution of -174 (G/C) IL-6 gene was not significantly different between the control subjects (GG 81.7%, GC 16.3%, CC 1.9%) and the AMI patients (GG 79%, GC 19%, CC 2%).The serum levels of IL-6 were significantly elevated in the AMI patients in comparison to the control subjects (*P* < 0.0001).

**Conclusions:**

There is no significant association of -174(G/C) polymorphism in the promoter sequence of IL-6 and the incidence of AMI in the examined sample of Egyptian population. Elevated levels of serum IL-6 confirmed the relationship between inflammation and the incidence of AMI.

## Background

Coronary artery disease (CAD) is the most common cause of mortality in the developed world [1]. It is caused by a blockage of the coronary arteries. The disease can cause myocardial ischemia and eventually necrosis of the heart [2]. It results from the collision of ancient genes with modern lifestyles: a hunter–gatherer lifestyle—with high daily energy expenditure and rare kills—favors a tendency to eat large quantities of high-calorie food when it is available. Such predispositions sit uneasily in a modern world with motorized transport and fatty snacks on every corner. Despite this, so-called “hardening of the arteries” was first described only in the 1700s, and it was not until the 1900s that a good description of myocardial infarction (MI) was presented [3]. MI is a multifaceted condition not based on a single factor or cause. In general, the incidence of MI increases additively as a function of the number of conventional risk factors, which include hypertension, diabetes mellitus, and hypercholesterolemia [4]. The constant accumulation of fat, immune cells, and fibrous tissues in the lining of the arteries can block the arteries. This can lead to decreased alertness, decreased blood flow, and eventually a CVD [5].

The term “CAD” encompasses a range of diseases that result from atheromatous change in coronary vessels stemming from genetic and environmental factors. Atherosclerosis is a cardiovascular disorder and a chronic inflammatory response by the body that has stable and unstable periods. This disorder occurs in the large and medium arteries. The cause of this disease is fat and cholesterol accumulation in the wall of the arteries [2]. In the past, CAD was thought to be a simple, inexorable process of artery narrowing, eventually resulting in complete vessel blockage. However, in recent years, the explanatory paradigm has changed because it was realized that a whole spectrum of coronary plaques exists—from stable (lipid-poor, thick fibrous cap) to unstable (lipid-rich, thin fibrous cap). Acute coronary artery syndrome (ACS), coronary artery disease (CAD), myocardial infarction (MI), stable and unstable angina, stroke, transient ischemic attack, and peripheral arterial disease are known as atherosclerotic subunits [6].

T and B lymphocytes as well as macrophages play a role in the development of atherosclerosis by secreting cytokines and other mediators [7]. Cytokines are also produced by other inflammatory cells, as well as vascular cells and adipocytes [8].

There are two types of cytokines, the pro-inflammatory cytokines TNF-α, IL-1, -6, -12, -15, -18, and -32, and the anti-inflammatory cytokines IL-10 and TGF-β. An autocrine activation loop in macrophages may involve self-stimulation by IL-12 and IL-18 to produce Interferon gamma (IFN γ )[8]. The human IL-6 gene is located on chromosome 7p21, and consists of 5 axons and 4 introns. IL-6 has several polymorphisms in the promoter region (-634 C/G, -174G/C, -572 G/C, and -597 G/A) [9].

Several reports described the genotype distribution of -174(G/C) polymorphism of the IL-6 gene among different population [10]. The IL-6 -174G allele had been demonstrated to be associated with higher IL-6 production. This polymorphism affects the circulating serum IL-6 level and IL-6 gene transcription. There have been extensive studies on IL-6 gene polymorphisms in different diseases and interestingly, there is also significant variation in the frequencies of this polymorphism among different ethnic groups [11]. It was reported that frequency of the -174C allele is much lower in the Japanese, Africans, and Asian Indians compared to European Caucasians [12].

Despite of these findings, no data regarding the -174(G/C) polymorphism distribution and its contribution to the incidence of cardiovascular diseases among Egyptians were available.

Accordingly, this study was designed to study the triangular relationship between the -174 G/C polymorphism, IL-6 serum concentrations, and the AMI. The assessment of this relationship required investigating the association between IL-6 -174 G/C polymorphism and the incidence of AMI in Egyptians, exploring the changes in the IL-6 serum concentrations in AMI, correlating the IL-6 gene variants to IL-6 serum concentrations to shed the light on the effect of this polymorphism on IL-6 synthesis and processing.

## Methods

### Study population

Random 100 AMI patients were recruited from the intensive care unit of the National Heart Institute. Patients were included if their clinical presentation, ECG, and cardiac biomarkers revealed acute single- or multi-vessel CAD. The individuals included 34 females and 66 males (age range 35 and 55 years). Data collection was done using a questionnaire asking about family, personal medical history, and health relevant behaviors. Exclusion criteria included individuals above the age of 55 years and those suffering from any acute or chronic severe diseases such as renal or hepatic insufficiency, diabetes mellitus, and other CVDs. Meanwhile, random 104 healthy volunteers attending the blood bank at 57357 Hospital in Cairo were enrolled in the study. They included 34 females (age range 18 and 52 years) and 70 males (age range 18 and 54 years). Questionnaires were used to obtain data about their family, personal history, and health-relevant behaviors, including exercise and diet. Exclusion criteria included any acute or chronic severe diseases such as renal or hepatic insufficiency, diabetes mellitus, and CVD. Informed written consent was obtained from all patients and healthy blood donors. The study is approved from the ethical committee of Faculty of Pharmacy and Biotechnology, German University in Cairo and complies with the ethics of Helsinki Declaration.

### Specimen collection

Blood samples were collected in both heparinized and non-heparinized tubes for whole blood and serum respectively. The serum samples were prepared by allowing the blood to clot at room temperature for 30 min and then centrifuged at 2500 rpm for 10 min at 4 °C. The serum was then frozen at − 80 °C until used for analysis. Samples were used to determine serum IL-6 levels. As for the whole blood, DNA was extracted, purified, and used for the genotype assay of -174(G/C) polymorphism of the IL-6 gene.

### Purification of DNA from human blood by spin protocol

Thermo Scientific GeneJET Whole Blood Genomic DNA Purification Mini Kit utilizes silica-based membrane technology was used. This provides DNA free of proteins, nucleases, and other contaminants and ready for use in the PCR. Samples were digested with Proteinase K in the supplied Lysis Solution. The lysate was then mixed with ethanol and loaded onto the purification column, where the DNA binds to the silica membrane. Impurities were effectively removed by washing the column with the prepared wash buffers. Genomic DNA was then eluted under low ionic strength conditions with the elution buffer.

### Screening for the G -174C variant in the promoter region of IL-6 gene

Genotyping was carried out using a newly designed primer pair to amplify the -174 G/C polymorphism. Briefly, DNA samples were amplified in polymerase chain reactions (PCR) with 10 pmol of both primers:

5′-TTGTCAAGACATGCCAAAGTG-3′ [sense]

5′-TCAGACATCTCCAGTCCTATA-3′ [antisense]

**Fig. 1:**
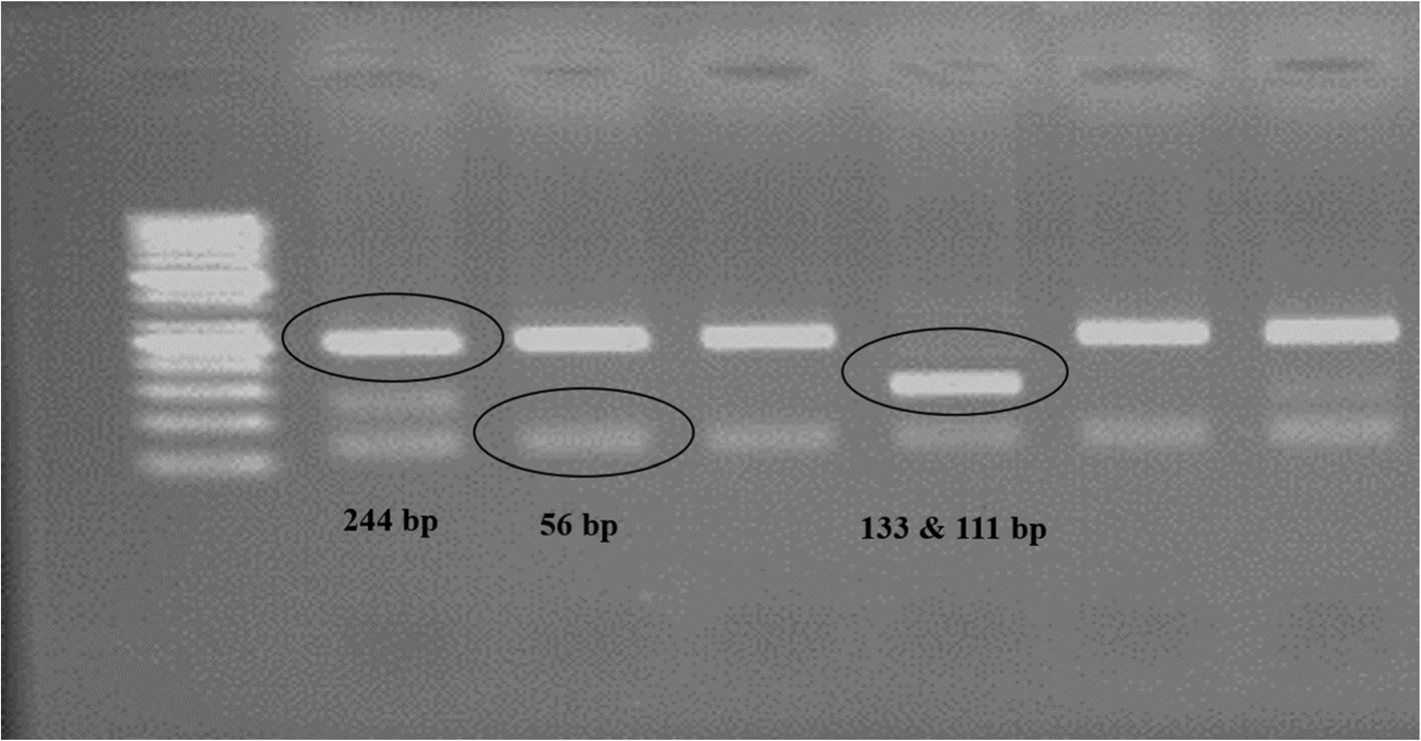
Representative of 2% agarose gel electrophoresis on NlaIII restriction of IL-6 gene from the blood of AMI patients. Lanes 1 and 6 heterozygote of GC; lanes 2, 3, and 5, homozygote of GG; lane 4, homozygote of CC

After PCR amplification, the fragments were digested with NlaIII restriction enzyme, followed by separation of the fragments on a 2% polyacrylamide gel. Fragments are visualized after staining with ethidium bromide. Due to one constant NlaIII restriction site the -174 G-allele (wild-type) had a size of 244 and 56 bp, and the -174 C-allele (mutant) had a size of 133, 111, and 56 bp, respectively (Fig. 1) [13].

### Assay of serum IL-6

Levels of serum IL-6 were determined quantitatively using Human IL-6/Interleukin-6 ELISA Kit PicoKine™ (Boster Biological Technology, Pleasanton CA, USA, Catalog # EK0410). A monoclonal antibody from mouse specific for IL-6 has been precoated onto 96-well plates. Standards (*E. coli*, P29-M212) and test samples are added to the wells, a biotinylated detection polyclonal antibody from goat specific for IL-6 is added subsequently and then followed by washing with phosphate buffered saline (PBS) buffer. Avidin-biotin-peroxidase complex was added and unbound conjugates were washed away with PBS buffer. Horseradish peroxidase (HRP) substrate 3,3′,5,5′ Tetramethylbenzidine (TMB) was used to visualize HRP enzymatic reaction. TMB was catalyzed by HRP to produce a blue color product that changed into yellow after adding acidic stop solution. The density of yellow is proportional to the human IL-6 amount of sample captured in plate.

### Statistical analysis

All statistical analyses were performed using the GraphPad prism statistics Software (GraphPad software, Inc.). Data are represented as median (interquartile range). Comparison of the differences between groups was done using the non-parametric Kruskal Wallis and the Mann-Whitney tests. A two-tailed *P* value ≤ 0.05 was considered statistically significant. Odds ratio is calculated by chi-square test.

## Results

### Serum levels of IL 6

**Fig. 2:**
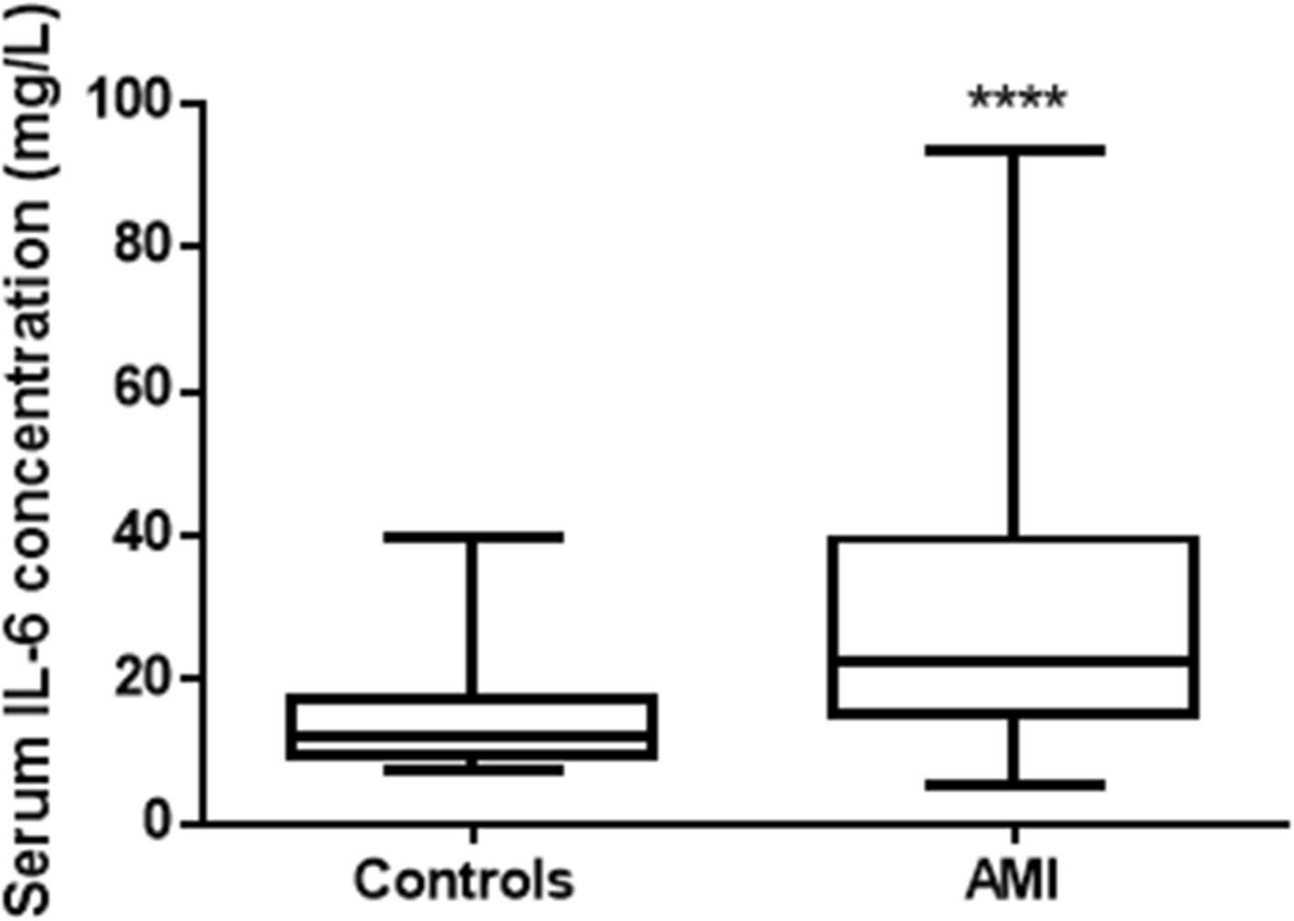
Boxplots showing serum median and interquartile levels (25th and 75th) of IL-6 concentration in control subjects and AMI. ***Significantly different at *P* < 0.0001 (Mann-Whitney test)

**Fig. 3:**
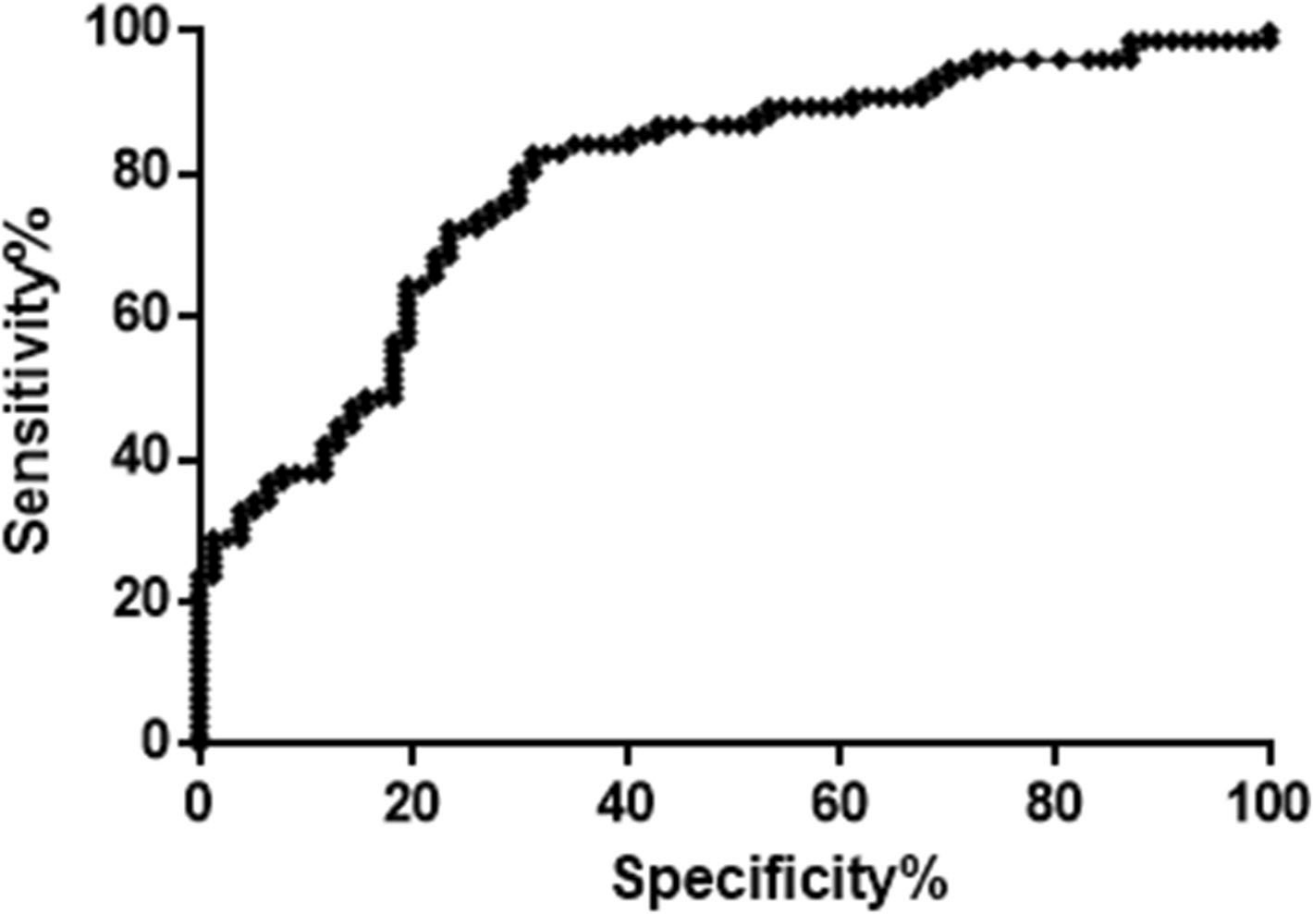
ROC curve of serum IL-6 concentrations

Serum levels of IL-6 in AMI patients (22.6 mg/L (15.4–39.5 mg/L)) showed significant increase up to 1.9 fold compared to the control group (12.0 mg/L(9.3–16.5)) (*P* < 0.0001, Mann-Whitney test) (Table 1, Fig. 2). ROC curve revealed that at a cutoff value 14.61 mg/L, the diagnostic sensitivity of IL-6 is 80% while its specificity is 71% (Fig. 3).

**Table 1:** Serum levels of IL6 in control and AMI groups

Groups	AMI	Control
Serum IL-6 (mg/L)	22.6 (15.4–39.5)	12.0 (9.3–16.5)

Results are expressed as median (interquartile range)

Mann-Whitney test

### Genotyping of -174 G/C variants of the IL-6 gene

**Fig. 4:**
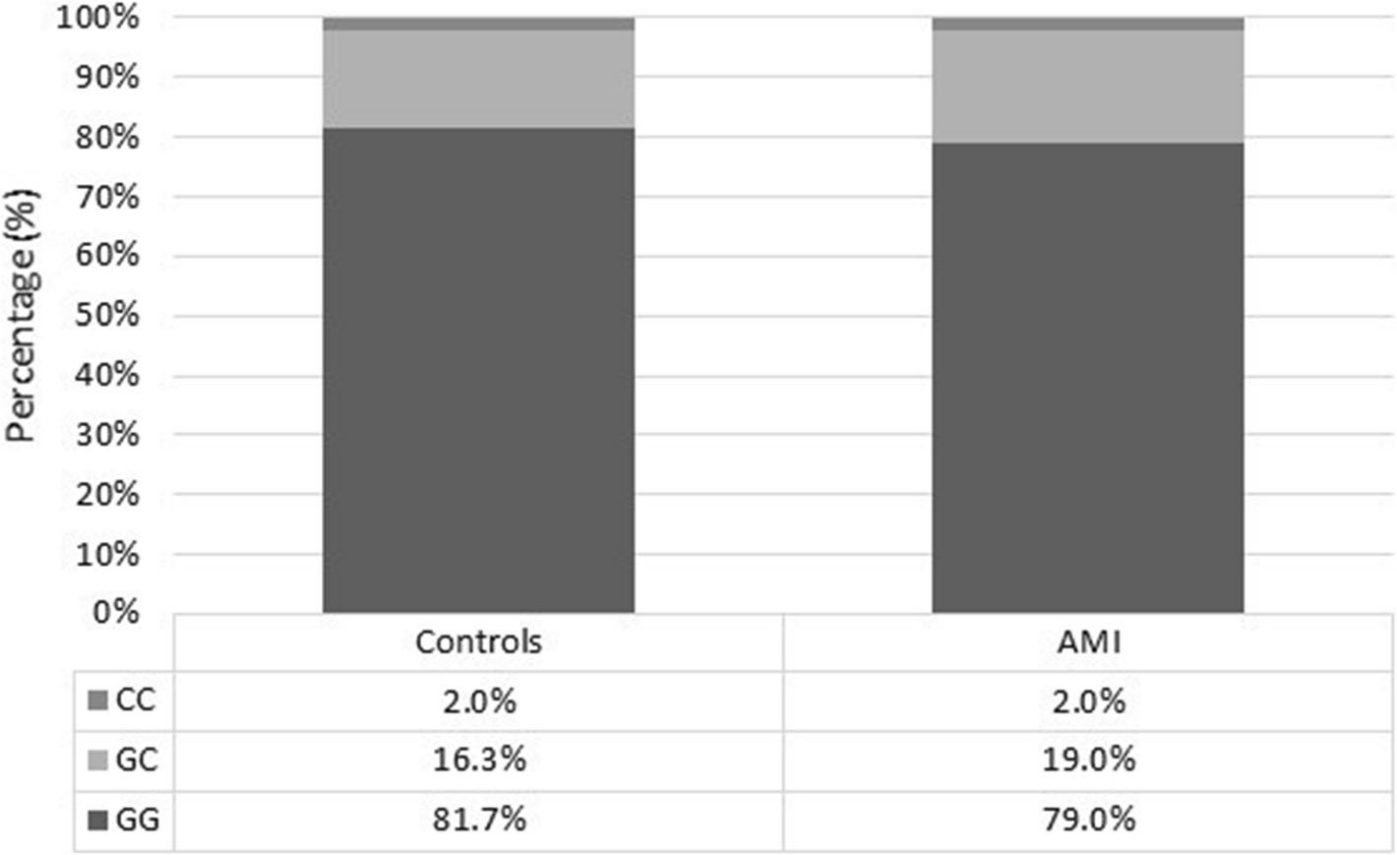
Percentage of subjects with each genotype in the control and AMI groups. There is no significant difference between the allele distribution between control and AMI subjects (*P* > 0.6376)

**Fig. 5:**
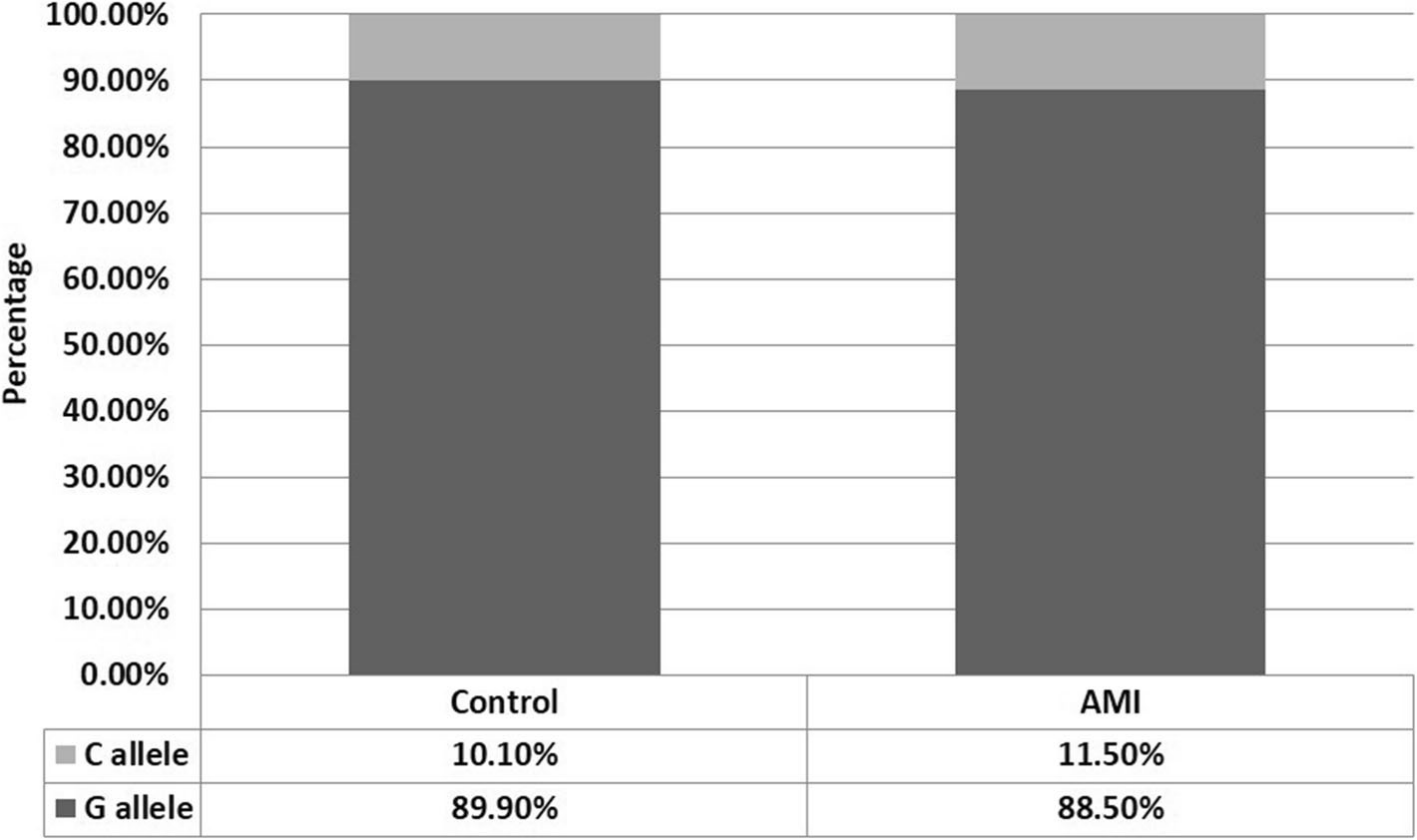
Allele frequencies of the IL-6 gene among control subjects and AMI patients

No significant association (*P* > 0.6213) was observed between the -174(G/C) polymorphism of the IL-6 and the incidence of AMI in the Egyptian population (Figs. 4 and 5). The genotype of the control subjects showed that the wild-type GG genotype was prevalent in 81.7% of the control subjects, while the GC and CC were present in 16.3% and 1.9% of the subjects, respectively. The allele frequencies of the G and C alleles were 89.9% and 10.1%, respectively. On the other hand, the genotype of the AMI patients revealed that wild-type GG was present in 79% of the patients, while the two other polymorphisms GC and CC were found in 19% and 2% of the subjects, respectively. The allele frequency of the G and C were 88.5% and 11.5%, respectively.

Odds ratio (OR) was calculated using SPSS by grouping CC + GC alleles versus GG allele (dominant model) and once grouping the GG + GC versus the CC allele (recessive model).

OR with its 95% confidence interval (CI) (Table 2). No deviation from Hardy-Weinberg equilibrium (HWE) was observed in the SNP’s genotype distribution (HWE *χ*2 = 1.40, *P* value = 0.236).

**Table 2:** Alleles and genetic models for IL-6 G174C SNP in AMI patients and controls

Allele/genotype(s)	AMI ***n*** (%)	Controls ***n*** (%)	OR (95% confidence interval)	***P*** value
**Alleles frequency**				
**G**	177 (88.5%)	187 (89.9%)	0.8642 (0.4619–1.617)	0.6477
**C**	23 (11.5%)	21 (10.1%)		
**Dominant model**				
**GG**	79 (79%)	85 (81.7%)	0.8409 (0.4208–1.680)	0.6234
**GC + CC**	21 (21%)	19 (18.3%)		
**Recessive model**				
**GG + GC**	98 (98%)	102 (98.1%)	0.9608 (0.1327–6.958)	0.9684
**CC**	2 (2%)	2 (1.9%)		

Chi-square test

No deviation from Hardy-Weinberg equilibrium (HWE) was observed in the SNP’s genotype distribution (HWE χ2 = 1.40, *P* value = 0.236; chi-square test)

### Association of IL-6 genotypes with the serum levels of IL-6

**Fig. 6:**
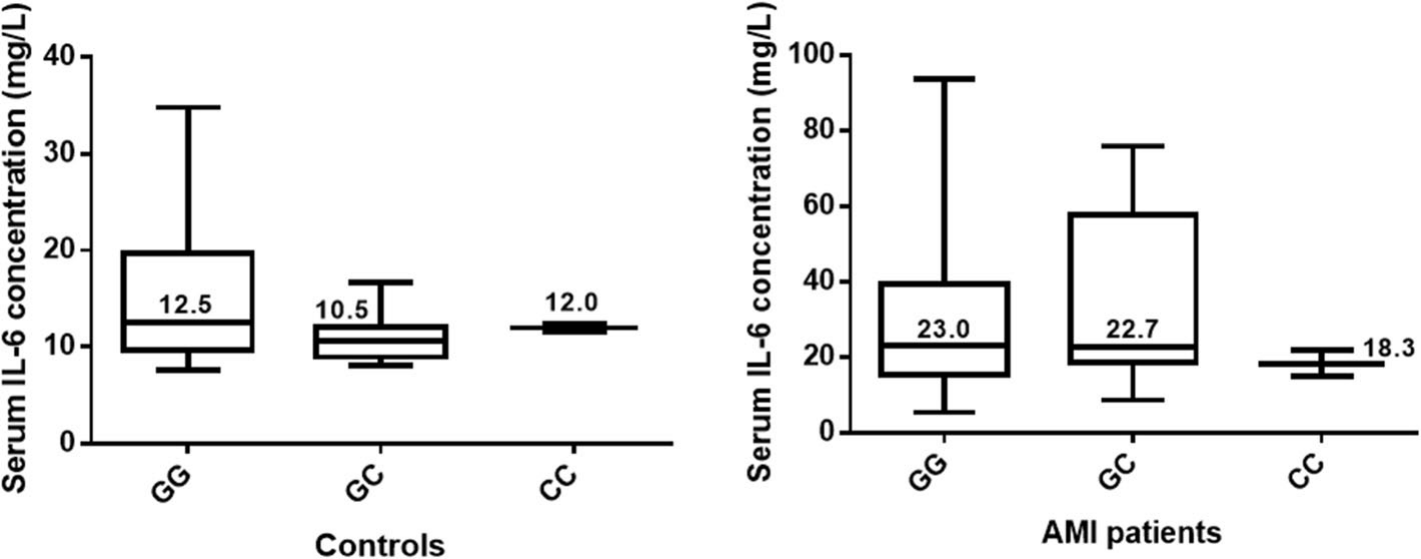
Boxplots showing serum median and interquartile levels (25th and 75th) of IL-6 concentration among different genotypes in controls and AMI patients

No significant difference was found among the serum IL-6 levels of the GG, GC, and CC genotypes in the AMI patients (medians GG = 23.0 mg/L; GC = 22.7 mg/L; CC = 18.3 mg/L) (*P* value = 0.5376; Kruskal-Wallis test) and controls (medians GG = 12.5 mg/L; GC = 10.5 mg/L; CC = 12.0 mg/L) (*P* value = 0.1401; Kruskal-Wallis test) (Fig. 6).

## Discussion

CAD is a major cause of death and disability in developed countries and it is responsible for about one third of all deaths in individuals over the age of 35 [4]. Genetics is considered to be one of the factors influencing the development of CAD. Some studies have reported 50 risk points in the human genome that can influence CAD development [14]. Extensive genomic studies demonstrate that genetic factors increase the chances of developing CAD by 1.1 to 1.3 times. It has also been shown that hereditary factors account for 30–60% of interpersonal differences in CAD [15].

Recently growing evidence has suggested that ongoing inflammation in the vessel wall accelerates progression of atherosclerosis and destabilizes the plaque. Plaque rupture causes atherothrombosis and subsequent MI [16].

Activated immune cells in the plaque produce inflammatory cytokines (interferon, interleukin-1, and tumor necrosis factor [TNF]), which induce the production of substantial amounts of interleukin-6.

### Genotype and allele distributions of -174G/C polymorphism of the IL-6 gene

The -174 G/C polymorphism has been inconsistently associated with AMI. In order to investigate this inconsistency, we performed this study which is the first clinical investigation done on the Egyptian population regarding the impact of the -174 G/C polymorphism on the incidence of CAD. No significant difference in genotypic or allelic distribution between AMI cases and controls was noticed.

Our results are similar to a study performed by Aker et al. that showed no significant differences in the allelic or genotypic frequencies between AMI patients and control. The genotype distribution and the allele frequencies of G -174 C polymorphism were similar in healthy controls (GG/GC 83%, CC 17%; G-allele 0.58, C-allele 0.42) and dialysis patients (GG/GC 86%, CC 14%; G-allele 0.61, C-allele 0.39) [17].

Another study supporting our results was done by Ghazouani et al. [18] who described the association of IL-6 promoter polymorphism -174 G/C with CAD in Tunisians. The main finding in their study was the lack of association of -174 G/C alleles or genotypes with CAD. The frequency of -174 C allele was comparable between CAD patients and healthy controls.

Furthermore, Sekuri et al. (2007) concluded that the IL-6 -174 G/C polymorphism is not associated with the risk of premature CAD, and does not contribute to cardiovascular risk stratification. The genotype distribution of the -174 G/C polymorphism was not different in premature CAD patients (GG 53%; GC 42.6%; CC 4.3%) and controls (GG 54.3%; GC 39%; CC 6.7%) (*P* = 0.72) [19].

Likewise, Lieb et al. reported that the IL-6 genotype was neither associated with traditional cardiovascular risk factors (systolic and diastolic blood pressure, total cholesterol, HDL and LDL cholesterol, body mass index, diabetes mellitus) nor with cardiac structural or functional parameters (left ventricular mass index, ejection fraction, diastolic inflow pattern). Moreover, the genotype distribution of the -174 G/C polymorphism was not different in MI patients (GG 34.1%; GC 47.4%; CC 18.5%) and population-based controls (GG 32.4%; GC 48.8%; CC 18.9%) (*P* = 0.67). IL-6 levels were not related to the -174 G/C polymorphism (*P* = 0.29) [20].

The current study results were also supported by a study conducted on 100 cases of CAD and 150 controls of Indo European descent from Maharashtra in Western India. They found no significant differences in the frequency of the IL-6 -174G > C genotypes between cases and controls [21].

In contrast, a study conducted on young South African Indian men concluded that the presence of the IL-6 -174 G allele influences levels of IL-6 and increases the risk of CAD in the tested population.

The difference in frequency was more pronounced when Indian controls were compared to black controls. A significant association between the -174 IL-6 G allele and CAD was found in Indian patients compared to Indian controls [22].

### Serum levels of serum IL-6

It has been observed that the serum levels of IL-6 are significantly higher in the AMI patients relative to the control subjects, which confirms the role of the inflammation in the incidence of AMI.

Supporting our results, Jenny et al. suggested that atherosclerosis represents a chronic inflammatory disorder and that elevated IL-6 levels may predict risk of future CVD events. In addition, polymorphisms in the 3′- and 5′-untranslated regions of the IL-6 gene may be key regulators of IL-6 and downstream protein levels and therefore may predispose an individual to CVD risk as well [23].

Similar to our results, Ikeda et al. have examined serum interleukin 6 (IL-6) levels in 12 patients with AMI. IL-6 levels became elevated in all patients, following the rise of serum creatine kinase (CK) activity [24].

An animal study showed that IL-6 mRNA was not detected in unstimulated “quiescent” rat cardiocytes cultured in serum-free medium, but its expression was induced by exposure of the cells to serum or ionomycin. These results show that IL-6 is synthesized in the myocardium and serum IL-6 levels become elevated in AMI, suggesting that IL-6 could affect the progression and/or healing processes of AMI [24].

Plasma concentrations of IL-6 are correlated to the severity of inflammation caused by fibrous plaques. Therefore, some studies suggested that IL-6 is a biomarker with better sensitivity and characteristics than CRP for the diagnosis of cardiovascular disease. A study by Lindmark et al. (2001) argue that IL-6 levels also play a role in mortality from heart disease [25].

On correlating the IL-6 levels to the genotypes, no significant difference in IL-6 concentration among different genotypes in both study groups. These results are similar to a study performed on Egyptian obese children which reported also that the IL-6 concentration was independent of -174 G/C in all study subjects [26]. Other studies also support this finding [27, 28]. However, some studies reported that GG genotype was associated with higher IL-6 levels [29, 30]. In contrast, another study reported that CC genotypes were associated with the highest levels [31]. These conflicting results might be attributed to differences in sample sizes, ethnicities, and studied diseases.

## Conclusions

In conclusion, the current study reported that no association was observed between the -174 G/C polymorphism of the IL-6 gene and the incidence of AMI in the studies Egyptian population. Patients having AMI had higher median serum levels of IL-6 when compared to healthy subjects confirming the role of inflammation in the incidence of AMI. However, there was a lack of association among the IL-6 genotypes and median serum level of IL-6 in AMI patients but association was found in control groups.

## Data Availability

The datasets used and/or analysed during the current study are available from the corresponding author on reasonable request.

## Abbreviations

ACS: Acute coronary artery syndrome
AMI: Acute myocardial infarction
CRP: C-reactive protein
CVD: Cardiovascular disease
HWE: Hardy-Weinberg equilibrium
IL: Interleukin
OR: Odds ratio
PCR-RFLP: Polymerase chain reaction-restriction fragment length polymorphism

## References

[1] Okrainec K, Banerjee DK, Eisenberg MJ (2004). Coronary artery disease in the developing world. Am Heart J..

[2] Salari N, Mansouri K, Hosseinian-Far A, Ghasemi H, Mohammadi M, Jalali R, Vaisi-Raygani A (2021). The effect of polymorphisms (174G> C and 572C> G) on the Interleukin-6 gene in coronary artery disease: a systematic review and meta-analysis. Genes Environ..

[3] Ashley EA, Niebauer J (2004) Cardiology Explained. London: Remedica. Chapter 5, Coronary artery disease. Available from: https://www.ncbi.nlm.nih.gov/books/NBK2216 20821845

[4] Hashad IM (2014). C242T polymorphism of NADPH oxidase p22phox gene reduces the risk of coronary artery disease in a random sample of Egyptian population. Mol Biol Rep.

[5] Maguire EM, Pearce SWA, Xiao Q (2019). Foam cell formation: a new target for fighting atherosclerosis and cardiovascular disease. Vascul Pharmacol.

[6] American Diabetes, A (2017). Cardiovascular Disease and Risk Management. Diabetes Care.

[7] Abdolmaleki F, Gheibi Hayat SM, Bianconi V, Johnston TP, Sahebkar A (2019). Atherosclerosis and immunity: a perspective. Trends Cardiovasc Med..

[8] Sprague AH, Khalil RA (2009). Inflammatory cytokines in vascular dysfunction and vascular disease. Biochem Pharmacol..

[9] Ryu JH, Kim SJ (2012). Interleukin-6 -634 C/G and -174 G/C polymorphisms in Korean patients undergoing hemodialysis. Korean J Intern Med..

[10] Gan GG, Subramaniam R, Lian LH, Nadarajan V (2013). Ethnic variation in interleukin-6 -174 (g/c) polymorphism in the malaysian population. Balkan J Med Genet..

[11] Trejaut JA, Tsai ZU, Lee HL, Chen ZX, Lin M (2004). Cytokine gene polymorphisms in Taiwan. Tissue Antigens..

[12] Ognjanovic S, Yamamoto J, Saltzman B, Franke A, Ognjanovic M, Yokochi L, Vogt T, Decker R, le Marchand L (2010). Serum CRP and IL-6, genetic variants and risk of colorectal adenoma in a multiethnic population. Cancer Causes Control..

[13] Zheng C, Huang DR, Bergenbrant S, Sundblad A, Österborg A, Björkholm M, Holm G, Yi Q (2000). Interleukin 6, tumour necrosis factor alpha, interleukin 1beta and interleukin 1 receptor antagonist promoter or coding gene polymorphisms in multiple myeloma. Br J Haematol..

[14] Hongmei Y, Yongping J, Jiyuan L (2016). Interleukin-6 polymorphisms and risk of coronary artery diseases in a Chinese population: a case-control study. Pak J Med Sci..

[15] Schunkert H, König IR, Kathiresan S, Reilly MP, Assimes TL, Holm H, Preuss M, Stewart AF, Barbalic M, Gieger C, Absher D, Aherrahrou Z, Allayee H, Altshuler D, Anand SS, Andersen K, Anderson JL, Ardissino D, Ball SG, Balmforth AJ, Barnes TA, Becker DM, Becker LC, Berger K, Bis JC, Boekholdt SM, Boerwinkle E, Braund PS, Brown MJ, Burnett MS, Buysschaert I, Carlquist JF, Chen L, Cichon S, Codd V, Davies RW, Dedoussis G, Dehghan A, Demissie S, Devaney JM, Diemert P, Do R, Doering A, Eifert S, Mokhtari NE, Ellis SG, Elosua R, Engert JC, Epstein SE, de Faire U, Fischer M, Folsom AR, Freyer J, Gigante B, Girelli D, Gretarsdottir S, Gudnason V, Gulcher JR, Halperin E, Hammond N, Hazen SL, Hofman A, Horne BD, Illig T, Iribarren C, Jones GT, Jukema JW, Kaiser MA, Kaplan LM, Kastelein JJ, Khaw KT, Knowles JW, Kolovou G, Kong A, Laaksonen R, Lambrechts D, Leander K, Lettre G, Li M, Lieb W, Loley C, Lotery AJ, Mannucci PM, Maouche S, Martinelli N, McKeown P, Meisinger C, Meitinger T, Melander O, Merlini PA, Mooser V, Morgan T, Mühleisen TW, Muhlestein JB, Münzel T, Musunuru K, Nahrstaedt J, Nelson CP, Nöthen MM, Olivieri O, Patel RS, Patterson CC, Peters A, Peyvandi F, Qu L, Quyyumi AA, Rader DJ, Rallidis LS, Rice C, Rosendaal FR, Rubin D, Salomaa V, Sampietro ML, Sandhu MS, Schadt E, Schäfer A, Schillert A, Schreiber S, Schrezenmeir J, Schwartz SM, Siscovick DS, Sivananthan M, Sivapalaratnam S, Smith A, Smith TB, Snoep JD, Soranzo N, Spertus JA, Stark K, Stirrups K, Stoll M, Tang WH, Tennstedt S, Thorgeirsson G, Thorleifsson G, Tomaszewski M, Uitterlinden AG, van Rij A, Voight BF, Wareham NJ, Wells GA, Wichmann HE, Wild PS, Willenborg C, Witteman JC, Wright BJ, Ye S, Zeller T, Ziegler A, Cambien F, Goodall AH, Cupples LA, Quertermous T, März W, Hengstenberg C, Blankenberg S, Ouwehand WH, Hall AS, Deloukas P, Thompson JR, Stefansson K, Roberts R, Thorsteinsdottir U, O'Donnell CJ, McPherson R, Erdmann J, Samani NJ, Cardiogenics, CARDIoGRAM Consortium (2011). Large-scale association analysis identifies 13 new susceptibility loci for coronary artery disease. Nat Genet..

[16] Hohda S, Kimura A, Sasaoka T, Hayashi T, Ueda K, Yasunami M, Okabe M, Fukuta N, Kurosawa T, Izumi T (2003). Association study of CD14 polymorphism with myocardial infarction in a Japanese population. Jpn Heart J..

[17] Aker S, Bantis C, Reis P, Kuhr N, Schwandt C, Grabensee B, Heering P, Ivens K (2009). Influence of interleukin-6 G-174C gene polymorphism on coronary artery disease, cardiovascular complications and mortality in dialysis patients. Nephrol Dial Transplant..

[18] Ghazouani L (2010). TNF-alpha -308G>A and IL-6 -174G>C polymorphisms in Tunisian patients with coronary artery disease. Clin Biochem.

[19] Sekuri C (2007). No association of interleukin-6 gene polymorphism (-174 G/C) with premature coronary artery disease in a Turkish cohort. Coron Artery Dis.

[20] Lieb W (2004). No association of interleukin-6 gene polymorphism (-174 G/C) with myocardial infarction or traditional cardiovascular risk factors. Int J Cardiol.

[21] Bhanushali AA, Contractor A, Das BR (2013). Variant at 9p21 rs1333049 is associated with age of onset of coronary artery disease in a Western Indian population: a case control association study. Genet Res (Camb)..

[22] Phulukdaree A, Khan S, Ramkaran P, Govender R, Moodley D, Chuturgoon AA (2013). The interleukin-6 -147 g/c polymorphism is associated with increased risk of coronary artery disease in young South African Indian men. Metab Syndr Relat Disord..

[23] Jenny NS (2002). In the elderly, interleukin-6 plasma levels and the -174G>C polymorphism are associated with the development of cardiovascular disease. Arterioscler Thromb Vasc Biol.

[24] Ikeda U, Ohkawa F, Seino Y, Yamamoto K, Hidaka Y, Kasahara T, Kawai T, Shimada K (1992). Serum interleukin 6 levels become elevated in acute myocardial infarction. J Mol Cell Cardiol..

[25] Ren G, Roberts AI, Shi Y (2011). Adhesion molecules: key players in mesenchymal stem cell-mediated immunosuppression. Cell Adh Migr..

[26] Ibrahim OM, Gabre AA, Sallam SF, el-Alameey IR, Sabry RN, Galal EM, Tawfik SM, Zarouk WA, Mosaad RM, Ramadan A (2017). Influence of Interleukin-6 (174G/C) Gene Polymorphism on Obesity in Egyptian Children. Open Access Maced J Med Sci..

[27] Rauramaa R, Väisänen SB, Luong LA, Schmidt-Trücksäss A, Penttilä IM, Bouchard C, Töyry J, Humphries SE (2000). Stromelysin-1 and interleukin-6 gene promoter polymorphisms are determinants of asymptomatic carotid artery atherosclerosis. Arterioscler Thromb Vasc Biol..

[28] Nauck M, Winkelmann BR, Hoffmann MM, Böhm BO, Wieland H, März W (2002). The interleukin-6 G(-174)C promoter polymorphism in the LURIC cohort: no association with plasma interleukin-6, coronary artery disease, and myocardial infarction. J Mol Med (Berl)..

[29] Jones KG, Brull DJ, Brown LC, Sian M, Greenhalgh RM, Humphries SE, Powell JT (2001). Interleukin-6 (IL-6) and the prognosis of abdominal aortic aneurysms. Circulation..

[30] Libra M, Signorelli SS, Bevelacqua Y, Navolanic PM, Bevelacqua V, Polesel J, Talamini R, Stivala F, Mazzarino MC, Malaponte G (2006). Analysis of G(-174)C IL-6 polymorphism and plasma concentrations of inflammatory markers in patients with type 2 diabetes and peripheral arterial disease. J Clin Pathol..

[31] Belluco C (2003). 174 G>C polymorphism of interleukin 6 gene promoter affects interleukin 6 serum level in patients with colorectal cancer. Clin Cancer Res.

